# Two-year outcomes of ThermoBlock radiofrequency ablation in chronic saphenous vein insufficiency: A single-center experience with 910 cases

**DOI:** 10.1016/j.jvsv.2025.102357

**Published:** 2025-11-21

**Authors:** Mehmet Emre Elci, Gözde Tekin

**Affiliations:** Cardiovascular Surgery Department, Dr Siyami Ersek Thoracic and Cardiovascular Surgery Education Research Hospital, Istanbul, Turkey

**Keywords:** Chronic venous insufficiency, Radiofrequency ablation, ThermoBlock, Venous Clinical Severity Score, VEINES-QOL/Sym, Endovenous thermal ablation, Long-term outcomes

## Abstract

**Background:**

Chronic venous insufficiency is a common vascular disorder that can lead to significant morbidity if left untreated. Endovenous thermal ablation, particularly radiofrequency ablation (RFA), has become a first-line therapy owing to its high efficacy and favorable safety profile. We sought to evaluate the technical success, safety, and clinical outcomes of the ThermoBlock RFA system in patients with reflux grade 4 great saphenous vein (GSV) insufficiency.

**Methods:**

This single-center retrospective study included 910 patients (444 males, 466 females; mean age, 45.4 ± 11.5 years) who underwent ThermoBlock RFA between March 2019 and June 2023. Clinical severity was assessed using the Venous Clinical Severity Score, and quality of life (QoL) was measured with the VEINES-QOL/Sym questionnaire. Duplex ultrasound examinations were performed at baseline and at 3, 6, 12, and 24 months. Statistical analyses used paired *t* tests for continuous variables and Wilcoxon signed-rank tests for ordinal variables, with effect sizes reported.

**Results:**

Technical success at 3 months was 97.6%. The Venous Clinical Severity Score improved from 10.9 ± 2.4 to 3.6 ± 0.9 (*P* < .001; Cohen's d = 3.7), and VEINES-QOL/Sym scores increased from 60.5 ± 7.71 to 84.6 ± 8.52 (*P* < .001; Cohen's d = 3.0). The mean GSV diameter decreased from 7.33 ± 1.13 mm before the procedure to 5.39 ± 0.60 mm at 3 months and 2.94 ± 0.66 mm at 24 months (*P* < .001). No mortality or pulmonary embolism occurred; deep vein thrombosis was observed in 0.7% of patients. Minor complications included thrombophlebitis (10.3%), hyperpigmentation (11.9%), ecchymosis (10.9%), and numbness (24.7%).

**Conclusions:**

ThermoBlock RFA is a safe and effective treatment for chronic GSV insufficiency, achieving high vein closure rates, substantial symptom relief, and improved QoL with minimal major complications. Its integrated laser guidance system may enhance procedural precision and support durable long-term outcomes.

**Clinical Relevance:**

Chronic venous insufficiency is a prevalent condition that significantly impairs QoL and imposes a substantial socioeconomic burden. Endovenous thermal ablation techniques have largely replaced conventional surgery owing to their high efficacy and lower complication rates. The ThermoBlock RFA system integrates precise thermal control with laser-guided catheter positioning, potentially enhancing procedural accuracy and outcomes. In this large, single-center study of 910 patients with advanced saphenous vein insufficiency, ThermoBlock RFA achieved a 97.6% closure rate at 3 months, with marked improvements in Venous Clinical Severity Score and VEINES-QOL/Sym scores, and a low incidence of major complications. These findings support ThermoBlock RFA as a safe, effective, and reproducible first-line option for the treatment of chronic venous insufficiency, even in patients with large-diameter great saphenous veins.

**Article Highlights**

• **Type of Research:** Retrospective single-center clinical study evaluating the 2-year outcomes of ThermoBlock radiofrequency ablation (RFA) in patients with reflux grade 4 great saphenous vein (GSV) insufficiency
• **Key Findings:** ThermoBlock RFA achieved a 97.6% technical success rate at 3 months, with sustained vein closure and progressive reduction in GSV diameter (from 7.33 mm to 2.94 mm at 24 months; *P* < .001). Significant improvements were observed in Venous Clinical Severity Score (from 10.9 ± 2.4 to 3.6 ± 0.9; *P* < .001) and VEINES-QOL/Sym scores (from 60.5 ± 7.7 to 84.6 ± 8.5; *P* < .001). No mortality or pulmonary embolism occurred; deep vein thrombosis developed in only 0.7% of patients. Minor complications were limited to thrombophlebitis (10.3%), hyperpigmentation (11.9%), ecchymosis (10.9%), and transient numbness (24.7%). Histological and ultrasound data confirmed durable anatomical remodeling and improved venous function.
• **Take Home Message:** ThermoBlock RFA is a safe, effective, and durable treatment for chronic GSV insufficiency. By combining real-time laser guidance with refined thermal control, it ensures high vein closure rates, significant symptom relief, and long-term quality-of-life improvement, with minimal complications. These findings position ThermoBlock as a reliable next-generation RFA system aligned with current European Society for Vascular Surgery 2022 and Society for Vascular Surgery/American Venous Forum 2023 venous treatment guidelines.

Chronic venous insufficiency is a common vascular disorder that affects 30% of the general population and causes leg pain, swelling, varicose veins, and skin changes; it can progress to ulcers in advanced stages.^1^ These advanced presentations, especially reflux grade 4 saphenous vein insufficiency, significantly impair quality of life (QoL) and increase the risk of complications such as deep vein thrombosis (DVT) and pulmonary embolism (PE), thereby placing a considerable burden on health systems.^2^

In line with the European Society for Vascular Surgery (ESVS) 2022 and Society for Vascular Surgery (SVS)/American Venous Forum (AVF) 2023 guidelines, reflux is defined as >500 ms in superficial truncal veins and >1 second in deep veins (with perforators ≥0.35-0.50 seconds). Because the guidelines do not mandate a universal four-grade classification, this study adopted an institutional duration-based grading system: grade 1 (0.5-1.0 second), grade 2 (1.0-2.0 seconds), grade 3 (2.0-3.5 seconds), and grade 4 (≥4.0 seconds on standing duplex ultrasound examination). This approach aligns with prior duration-based classifications and classic evidence showing that longer reflux times correlate with greater severity of venous insufficiency.^3^^,^^4^

In recent decades, endovenous thermal ablation (EVTA) techniques, particularly radiofrequency ablation (RFA), have replaced open surgery as the treatment of choice owing to their minimally invasive nature, high occlusion rates, and lower complication profiles. RFA achieves vein closure by applying controlled thermal energy to induce endothelial damage, collagen contraction, and subsequent fibrosis.^5^ Meta-analyses and randomized controlled trials have shown that RFA has comparable or superior short- and long-term outcomes relative to endovenous laser ablation, with fewer instances of pain, ecchymosis, paresthesia, and recurrence, particularly in studies conducted since 2016.^6^

The ESVS and AVF guidelines endorse RFA as a first-line treatment for superficial venous insufficiency (Clinical, Etiological, Anatomical, Pathological [CEAP] C2-C6), emphasizing its favorable risk-benefit profile.^7^ Randomized trials and long-term data further support the sustained efficacy and QoL improvements with endothermal approaches, showing durability up to 10 years in HELP (A New Method of Surgically Treating Varicose Veins and Venous Ulcers - a Study to Assess Clinical and Economic Value).^8^

Advancements in RFA technology—like the ThermoBlock system—offer enhanced catheter design, real-time visualization, and refined thermal control, which may improve procedural accuracy and safety. However, large-scale, long-term data on this device are scarce.^9^ Therefore, the present study aimed to evaluate the technical success, safety, and both early and long-term clinical and anatomical outcomes of ThermoBlock RFA in patients with reflux grade 4 great saphenous vein (GSV) insufficiency. To evaluate clinical and patient-reported outcomes, two validated instruments were used. The Venous Clinical Severity Score (VCSS) provides a standardized assessment of disease severity based on objective clinical parameters such as pain, varicose veins, edema, pigmentation, and ulceration. The VEINES-QOL/Sym questionnaire is a disease-specific tool designed to measure symptom burden and QoL in patients with chronic venous disorders. Both instruments are widely endorsed in current venous guidelines and allow consistent comparison of therapeutic outcomes across studies. We assessed results using duplex ultrasound examination for anatomical closure, the VCSS for disease severity, and the VEINES-QOL/Sym questionnaire for disease-specific QoL.^10^, ^11^, ^12^

## Methods

### Study design and patient selection

The study protocol was approved by the Clinical Research Ethics Committee of Haydarpaşa Numune Training and Research Hospital (Approval No: HNEAH-KAEK 2022/196-3907) and complied with the principles of the Declaration of Helsinki. All participants provided written informed consent prior to inclusion, in accordance with Good Scientific Practice standards.

This retrospective single-center study was conducted between March 2019 and June 2023. Medical records of 1468 patients diagnosed with reflux grade 4 venous insufficiency and a GSV diameter of >5.5 mm were reviewed. A total of 910 eligible patients (444 males, 466 females) with complete clinical and imaging data were included (Table I). Exclusion criteria were a history of venous thrombosis, active infection or ulcerated lesion, deep venous insufficiency, and malignancy. Only one limb was treated per session. Incompetent perforator veins were treated by open subfascial ligation during the same session. The ThermoBlock RF catheter was used exclusively for ablation of the GSV, and a perforator-specific RF catheter was not applied, primarily because such devices are not routinely available in our institution owing to cost considerations. Therefore, open ligation was adopted as the standard approach for managing perforators in this study. When necessary, concomitant miniphlebectomy was performed in patients with varicose clusters. Written informed consent was obtained from all patients, with detailed explanation of potential risks including postoperative numbness, phlebitis, sensory loss, thrombosis, ecchymosis, DVT, and PE.

**Table I:** Preprocedural sociodemographic and clinical features of the patients (n = 910)

Characteristics	
Age, years	45.4 ± 11.5 (20-76)
BMI	28.98 ± 1.71 (25.2-32.9)
Sex	
Male	444 (48.8)
Female	466 (51.2)
Smoking	
No	371 (40.8)
Yes	539 (59.2)
Hypertension	
No	811 (89.1)
Yes	99 (10.9)
Diabetes mellitus	
No	727 (79.9)
Yes	183 (20.1)
CEAP class	
1	195 (21.4)
2	317 (34.8)
3	271 (29.8)
4	112 (12.3)
5	15 (1.6)
Reflux grade-4	910 (100)

*BMI,* Body mass index; *CEAP,* Clinical, Etiological, Anatomical, Pathological.

Values are mean ± standard deviation (min-max) or number (%).

### Preoperative assessment

Preoperative evaluation included physical examination and duplex ultrasound examination performed in the standing position using distal augmentation-release or Valsalva at the saphenofemoral junction and thigh segments, in line with guidelines, to measure reflux times and GSV diameters. According to current guidelines, reflux is defined as >500 ms of reversed flow in superficial truncal veins (GSV/small saphenous vein and accessory branches), >1.0 second in the deep system (common femoral vein/femoral vein/popliteal vein), and ≥0.35 to 0.50 seconds in perforators.^3^^,^^4^ The evaluated perforators included the Cockett (posterior tibial), Boyd (paratibial), Dodd (distal thigh), and Hunterian (proximal thigh) perforators, which are the most clinically relevant channels in GSV insufficiency.

These thresholds define the presence of reflux; a universal four-grade severity scale is not mandated by guidelines. Because the guidelines do not mandate a universal four-grade scheme, we specified our institutional, duration-based grading. Building on the guideline thresholds and a duration-based concept used in prior research (longer reflux = higher severity), we categorized reflux as follows: grade 1, 0.5 to 1.0 second; grade 2, 1.0 to 2.0 seconds; grade 3, 2.0 to 3.5 seconds; and grade 4 (severe), ≥4.0 seconds. This approach aligns with prior duration-based classifications and with classic evidence that longer times correlate with severe reflux.^13^, ^14^, ^15^, ^16^ Clinical severity was assessed using the VCSS, and disease-specific QoL was measured using the VEINES-QOL/Sym questionnaire (Turkish validated versions). Follow-up duplex ultrasound evaluations were scheduled at baseline, 3 months, 6 months, 12 months, and 24 months post procedure.

### RFA procedure

**Fig 1:**
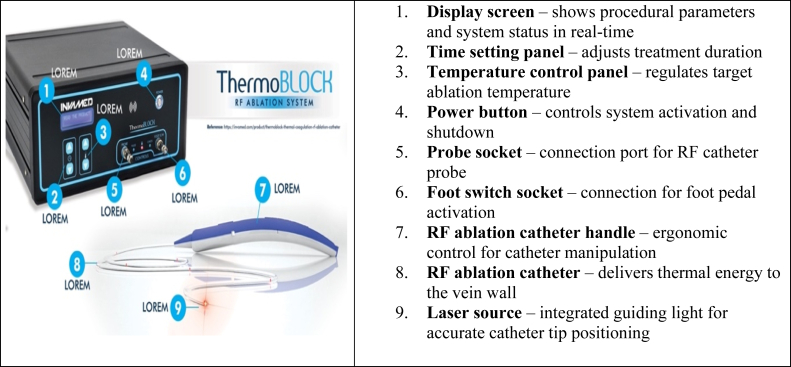
Main components of the ThermoBlock radiofrequency ablation (RFA) system. (Obtained kindly from Invamed, Ankara, Turkey.)

All procedures were performed under spinal anesthesia by two same experienced cardiovascular surgeons at the same center using a single-use ThermoBlock RFA system (Invamed) (Fig 1).

1. Under ultrasound guidance, the GSV was punctured below the knee, and a 7F introducer sheath was advanced.
2. The ThermoBlock RFA catheter was positioned 2 cm distal to the saphenofemoral junction. Positioning was confirmed by both Doppler ultrasound examination and red light transillumination.
3. Tumescent anesthesia (500 mL cold isotonic solution + 40 mL bicarbonate + 0.5 mg epinephrine + 5 mg prilocaine) was infiltrated along the vein to protect surrounding tissues from thermal injury.
4. Thermal energy was delivered at 10 J per 1 mm vein diameter, with continuous slow pullback at 1 mm/second and gentle manual compression.
5. Anatomical variations (double GSV, accessory GSV) were addressed with additional punctures, and significant residual perforators were ligated subfascially in the same session.
6. Post procedure, patients were monitored overnight, received single dose of 4000 IU factor Xa inhibitor.
7. All patients were systematically evaluated for endovenous heat-induced thrombosis (EHIT) by duplex ultrasound examination an additional early assessment was performed before discharge after routine overnight hospitalization.
8. All patients received venoactive medication (diosmin/hesperidin-based agents) and compression stockings for 3 months postoperatively as part of our institutional standard protocol.

### Study end points

The primary objectives of our study were as follows.

1. Technical success, defined as complete occlusion of the treated vein on duplex ultrasound examination, with no residual flow >10 cm in length. A decrease in vein diameter was recorded during follow-up, but this was considered a supportive parameter rather than the primary definition of technical success. This definition is consistent with prior EVTA trials and current guideline recommendations
2. Clinical efficacy was defined as a significant improvement in validated clinical scores, specifically the VCSS and the VEINES-QOL/Sym score. These were systematically assessed at baseline and during follow-up. The VSymQ instrument was not used in our cohort. Improvement in either score was considered supportive evidence of efficacy; however, our primary definition of clinical success relied on concordant improvements in both VCSS and VEINES-QOL/Sym across the cohort. This approach is consistent with the 2022 ESVS guidelines, which recommend the use of standardized clinical and QoL measures such as VCSS and validated patient-reported outcome tools when evaluating treatment efficacy in chronic venous disease.
3. Safety, measured by perioperative and postoperative complications (EHIT, thromboembolic events, nerve injury, burns, infection).

Secondary objectives included symptomatic relief, the need for adjunctive procedures (phlebectomy or perforator ligation), Durability of outcomes (recanalization or recurrent reflux at 3 and 12 months and 24 months), and recovery parameters such as hospital stay and return to normal activities. Duplex ultrasound outcomes were recorded at 1 week, 1 month, 3 months, 12 months, and 24 months. Scores were systematically assessed at 1 week, 1 month, 3 months, and 12 months after the procedure. Owing to an inadequate number of patients at the 12-month follow-up, the scores were not considered in the final analysis. This framework allowed a comprehensive evaluation of both efficacy and safety.

### Statistical analysis

The data were analyzed using IBM SPSS Version 21 and MedCalc statistical package program. Continuous variables are reported as mean ± standard deviation together with 95% confidence intervals (CIs) for mean differences; categorical variables are presented as number (%). In line with the central limit theorem,^17^ parametric tests were applied to continuous data; VEINES-QOL/Sym and GSV diameters were compared using paired *t* tests, with Cohen's dᶻ reported as effect size for paired designs. The VCSS, being ordinal, was analyzed using the Wilcoxon signed-rank test, and effect size was expressed as r = Z/√N. A two-tailed *P* value of <.05 was considered statistically significant.

## Results

### Patient demographics and baseline characteristics

**Fig 2:**
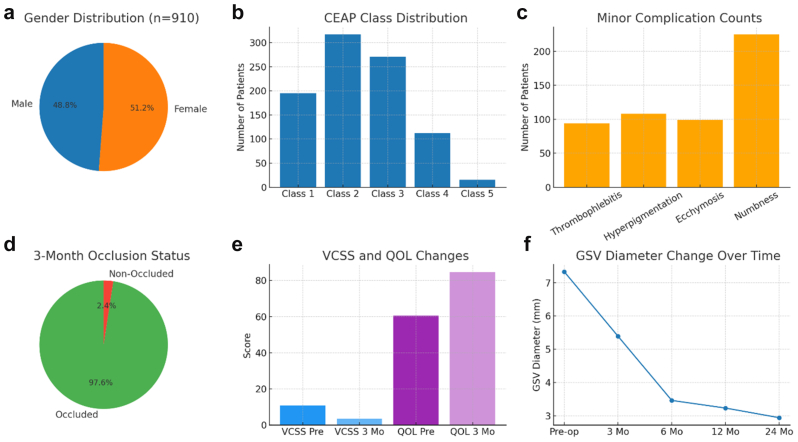
Graphical representation of patient demographics, disease classification, clinical outcomes, and procedural results. **(A)** Gender distribution of the study cohort (n = 910). **(B)** Clinical, Etiological, Anatomical, Pathological (*CEAP*) class distribution at baseline. **(C)** Distribution of minor complications observed during follow-up, including thrombophlebitis, hyperpigmentation, ecchymosis, and numbness. **(D)** Occlusion status at the month 3 duplex ultrasound (DUS) follow-up examination. **(E)** Preprocedural and postprocedural changes in Venous Clinical Severity Score (*VCSS*) and VEINES-QOL/Sym scores, both demonstrating significant improvement (*P* < .001). **(F)** Temporal change in great saphenous vein (*GSV*) diameter from baseline to 24 months, with the most prominent reduction occurring in the early postoperative period.

A total of 910 patients (444 males [48.8%]; 466 females [51.2%]) (Fig 2, *A*) with a mean age of 45.4 ± 11.5 years (range, 20-76 years) and a mean body mass index of 28.98 ± 1.71 kg/m^2^ (range, 25.2-32.9 kg/m^2^) were included in the study (Table I). Smoking was reported in 59.2% of patients, hypertension in 10.9%, and diabetes mellitus in 20.1%. According to CEAP classification, the majority were in class 2 (34.8%) and class 3 (29.8%), followed by class 1 (21.4%), class 4 (12.3%), and class 5 (1.6%). All patients had reflux grade 4 at baseline (Table I and Fig 2, *B*).

### Technical success and safety outcomes

At the 3-month follow-up, duplex ultrasound examination confirmed successful vein occlusion in 888 patients (97.6%; 95% CI, 96.3-98.6), and 22 patients (2.4%) remained nonoccluded (Table II). No procedure-related mortality occurred. No PE was observed in any patient, and DVT developed in six patients (0.7%, 95% CI, 0.3-1.5). Minor complications included thrombophlebitis in 94 patients (10.3%), hyperpigmentation in 108 patients (11.9%), ecchymosis in 99 patients (10.9%), and numbness in 225 patients (24.7%) (Table II and Fig 2, *C*).

**Table II:** Rates of postprocedural occlusion and adverse events (n = 901 patients)

Postprocedural observation	No.	%
Status of occlusion (month 3)		
Nonoccluded	22	2.4
Occluded	888	97.6
Adverse event		
DVT		
No	904	99.3
Yes	6	0.7
Pulmonary emboli		
No	910	100
Yes	0	0
Thrombophlebitis		
No	816	89.7
Yes	94	10.3
Hyperpigmentation		
No	802	88.1
Yes	108	11.9
Ecchymosis		
No	811	89.1
Yes	99	10.9
Numbness		
No	685	75.3
Yes	225	24.7

*DVT,* Deep vein thrombosis.

### Clinical and QoL outcomes

The VCSS score improved significantly from 10.9 ± 2.4 at baseline to 3.6 ± 0.9 at month 3 (*P* < .001; Cohen's d = 3.7), indicating a large treatment effect (Table III and Fig 2, *D*). Similarly, the VEINES-QOL/Sym score increased from 60.5 ± 7.71 to 84.6 ± 8.52 (*P* < .001; Cohen's d = 3.0), reflecting a substantial QoL improvement (Table III and Fig 2, *E*).

**Table III:** Comparison of Venous Clinical Severity Score (*VCSS*), VEINES-QOL/Sym, and great saphenous vein (*GSV*) diameter at preprocedural and the follow-up periods

	Preprocedural	Month 3	Month 6	Month 12	Month 24	*P* value
VCSS	10.9 ± 2.4	3.6 ± 0.9	-	-	-	<.001
VEINES-QOL/Sym	60.5 ± 7.71	84.6 ± 8.52	-	-	-	<.001
GSV diameter, mm	7.33 ± 1.13	5.39 ± 0.6	3.46 ± 0.58	3.23 ± 0.63	2.94 ± 0.66	

*P* values were considered significant when <.5.

Because of the 97.6% occlusion rate, VCSS and QoL were not followed up after 3 months.

Values are mean ± standard error (95% confidence interval).

### Ultrasound vessel diameter changes

The mean preoperative GSV diameter was 7.33 ± 1.13 mm. The estimated mean diameter at month 3 was 5.39 ± 0.60 mm, followed by further decreases at 6 months (3.46 ± 0.58 mm), 12 months (3.23 ± 0.63 mm), and 24 months (2.94 ± 0.66 mm) (*P* < .001 for all time points compared with baseline) (Table III and Fig 2, *F*). The most pronounced reduction occurred within the first 3 months, with a gradual decrease thereafter.

## Discussion

Chronic venous insufficiency is a progressive disorder that, if left untreated, can lead to complications such as venous ulcers and venous thromboembolism, imposing a considerable burden on health care systems worldwide.^18^ Treatment selection should therefore be guided by both short- and long-term evidence regarding occlusion rates, symptom improvement, QoL, and safety.^19^

EVTA has largely replaced conventional surgery as the first-line therapy for saphenous vein incompetence because of its high success rates, lower morbidity, and faster recovery.^20^ Among EVTA modalities, RFA offers precise thermal energy delivery with less perivenous injury and discomfort.^21^

The ThermoBlock RFA system applies controlled heat ≤120°C to induce collagen denaturation and fibrotic occlusion of the treated vein. Its integrated real-time laser guidance system enhances catheter positioning and energy control, representing a refinement not available in earlier RFA technologies. Beyond this technical advancement, our study provides clinical novelty by reporting one of the largest single-center RFA cohorts to date (910 patients) with follow-up extending to 24 months, offering robust long-term data rarely documented in the literature. ThermoBlock achieved a high technical success rate (97.6%), significant clinical improvements in both VCSS and VEINES-QOL/Sym scores (*P* < .001 for both), with effect sizes (Cohen's d > 3.0), and maintained an excellent safety profile (0.7% DVT, no PE or mortality).^22^, ^23^, ^24^, ^25^ Our study also documented a marked reduction in GSV diameter—from 7.33 mm before the procedure to 5.39 mm at 3 months, further decreasing to 2.94 mm at 24 months (*P* < .001). The progressive and sustained decrease in GSV diameter over 24 months further indicates durable anatomical remodeling. Taken together, these findings suggest that ThermoBlock combines meaningful technological refinement with strong long-term clinical validation, contributing valuable evidence to the ongoing evolution of RFA therapy.^26^

Although procedures were performed under spinal anesthesia, we deliberately used ultrasound-guided perivenous tumescent anesthesia containing prilocaine. Tumescent infiltration provides complementary local analgesia, and randomized data show that buffered tumescent solutions significantly decrease perioperative and early postoperative pain during endothermal ablation.^19^ It also functions as a heat-sink and hydrodissection layer, reducing perivenous temperatures, protecting skin and adjacent nerves from thermal injury, and improving catheter-vein contact.^27^ Furthermore, by compressing the vein and evacuating blood, tumescence aids hemostasis and procedural efficacy.^28^ Regarding the agent, prilocaine has pharmacokinetics comparable with those of lidocaine and has been reported as effective and safe in tumescent formulations for EVTA/RFA.^29^

In our institution, spinal anesthesia and overnight monitoring were routine for safety—allowing early detection of hematoma, DVT, or anesthesia-related effects—and were not study specific. Similarly, a single 4000 IU dose of a factor Xa inhibitor was administered as routine thromboprophylaxis given the large sample size, spinal anesthesia, and frequent adjunctive procedures. This practice is supported by Braet et al,^30^ who showed that standardized VTE prophylaxis reduced DVT/PE incidence after superficial venous interventions. No EHIT cases were detected during follow-up, consistent with ESVS 2022 and SVS/AVF 2023 recommendations for prophylaxis in higher-risk patients.^3^^,^^4^

Minor complications included superficial thrombophlebitis (10.3%), hyperpigmentation (11.9%), ecchymosis (10.9%), and transient numbness (24.7%), defined as a temporary sensory change recorded at early follow-up. Although this rate appears to be higher than in most RFA series (commonly 2%-10%), it primarily reflects patients who underwent concomitant perforator ligation or varicose cluster excision in addition to ThermoBlock RFA. These adjunct surgical procedures are well-known to increase the risk of transient sensory disturbances.^31^^,^^32^ In contrast, patients treated with RFA alone demonstrated numbness rates comparable with contemporary reports,^24^ and most symptoms resolved within 3 months. Therefore, the observed neurological findings should not be attributed solely to the ThermoBlock system itself, but rather to the inclusion of patients undergoing combined procedures. Overall, the safety outcomes of ThermoBlock remain consistent with modern RFA literature.

Concomitant miniphlebectomy and perforator ligation were performed when indicated, although results were analyzed as a single cohort. These additional procedures may increase thromboembolic and neurological events, as previously reported.^30^ Nevertheless, our primary outcomes—technical success and clinical improvement—remained robust. Future prospective studies comparing RFA-only and combined approaches would clarify respective safety profiles.

All patients were prescribed venoactive medication (diosmin/hesperidin 500 mg) and compression stockings for 3 months postoperatively, per institutional protocol. Because this regimen was applied uniformly and all patients had previously failed conservative therapy, the observed improvements can be attributed primarily to ThermoBlock RFA rather than adjunctive treatments. Randomized trials have similarly shown that endovenous ablation provides superior and durable outcomes compared with compression therapy alone.^33^, ^34^, ^35^

ThermoBlock demonstrated high efficacy, durability, and safety in a large cohort, aligning with and extending current evidence for RFA. The combination of advanced catheter design and robust long-term results supports its role as a reliable next-generation endothermal ablation system.

## Study limitations

The main limitation of this study is its single-center, retrospective, and noncontrolled design, with limited long-term VCSS and QoL assessments beyond three months owing to the high early occlusion rate. Nevertheless, the inclusion of 910 patients—one of the largest single-center RFA cohorts reported to date—with ≤24 months of follow-up provides strong clinical evidence that extends beyond typical retrospective studies. The combination of objective duplex ultrasound findings and substantial improvements in validated clinical scores supports the reliability of the results. Despite these strengths, future prospective multicenter trials with extended follow-up are warranted to confirm these findings and further evaluate the long-term durability of ThermoBlock outcomes.

## Conclusions

The ThermoBlock RFA system represents a safe and effective modality for the treatment of chronic saphenous vein insufficiency, achieving high vein closure rates, significant clinical and QoL improvements, and a low incidence of major complications. The system's integrated real-time laser guidance may enhance catheter precision and energy control, potentially contributing to its high technical success and favorable safety profile.

## Author contributions

Conception and design: ME, GT

Analysis and interpretation: ME, GT

Data collection: ME, GT

Writing the article: ME

Critical revision of the article: ME, GT

Final approval of the article: ME, GT

Statistical analysis: GT

Obtained funding: Not applicable

Overall responsibility: GT

## Funding

None.

## Disclosures

None.
